# Supplementary material for: Acute Kidney Injury in a Cohort of Critical Illness Patients Exposed to Non-Steroidal Anti-Inflammatory Drugs
Source: Pharmaceuticals (Basel). 2022 Nov 14;15(11):1409. doi: 10.3390/ph15111409 (PMC9693114; doi:10.3390/ph15111409)
Supplement: Supplementary file 1 [file pharmaceuticals-15-01409-s001.zip › pharmaceuticals-1998723-supplementary.pdf]

Table S1. Dimensions taken into account in the construction of the algorithms

COMORBIDITY	ICD-10 CODES	CUPS AND MEDICATION
MYOCARDIAL INFARCTION	I21* I22* I252 I255	360100 360101 360102 360102 360200 360201 360202
CONGESTIVE HEART FAILURE	I110 I130 I132 I50* I420 I425 I429 I43*	
PERIPHERAL VASCULAR DISEASE	K551 K558 K559 Z958 Z959 I70* I71* I720 I728 I739 I719 I731 I738 I771 I790 I792	380300 380910 383903 392204 392400 392501 392502 392503 392601 392602 392603 392604 392605 380300 392620
STROKE	G45* G46* I60* I69* I67* I681 I682 I688 I694 I698	395010 380101 380110 380210
DEMENTIA	F00* F01* F02* FO3* G30* G311 F03X F051 F010	*RIVASTIGMINE* *DONEPEZIL* *GALANTAMINE* *MEMANTINE* *DONEPEZIL MEMANTINE* *BUPROPION* *AMANTADINE*
CHRONIC OBSTRUCTIVE PULMONARY DISEASE	J441 I278 I279 J4* J60* J61* J62* J63* J64* J66* J67* J684 J701 J703 J448 J449 J410 J411 J42X J431 J432 J438 J439	*OXYGEN*
CONNECTIVE TISSUE DISEASE	M053 M058 M059 M060 M061 M063 M069 M050 M052 M051 M353 M320 M321 M328 M329 M353 M330 M331 M332 M339 M340 M341 M342 M348 M349 M353	*METOTREXATE* *SULFASALAZINE* *LEFLUNOMIDE* *PENICILAMINE* *RITUXIMAB* *ABATACEPT* *ETANERCEPT* *INFLIXIMAB* *ADALIMUMAB* *CERTOLIZUMAB* *GOLIMUMAB* *TOCILIZUMAB* *TOFACIAXIN* *ANAKINRA* *TACROLIMUS* *ACTEMRA*
PEPTIC ULCER DISEASE	K270 K271 K272 K273 K274 K275 K276 K279 K250 K251 K252 K253 K254 K255 K256 K257 K259 K260 K261 K262 K263 K264 K265 K266 K267 K269	S22224 441100 451301 451600 893904 901220 906022 906023 906024 438100 440100
MILD LIVER DISEASE	K709 K702 K703 K717 K740 K742 K746 K740 K742 K746 K743 K744 K745 K730 K731 K738 K739 k70* k73*	
TYPE 2 DIABETES	E100 E101 E106 E108 E109 E110 E111 E116 E118 E119 E120 E121 E126 E128 E29 E130 E131 E136 E138 E139 E140 E141 E146 E148 E149 E140	*REPAGLINIDE* *NATEGLINIDE* *LIRAGLUTIDE* *EXENATIDE* *LIXIZENATIDE* *DULAGLUTIDE* *ACARBOSE* *MIGLITOL* *CLORPROPAMIDE* *TOLBUTAMIDE* *GLIBENCLAMIDE* *GLIMEPIRIDE* *GLICLAZIDE* *GLIBENS* *GLIPIZIDE* *INSULIN* *INSUMAN* *INSULEX* *INSULIN ASPART* *INSULIN GLULISINE* *INSULIN LISPRO* *INSULIN DETEMIR* *INSULIN GLARGINE* *INSULIN DEGLUDEC* *INSULIN NPH* *METFORMIN* *GEMIGLIPTIN* *EMPAGLIFLOZIN* *PHENFORMIN* *SITAGLIPTIN* *VILDAGLIPTIN* *LINAGLIPTIN* *ALOGLIPTIN*
PARAPLEGIA - HEMIPLEGIA	G81 G041 G820 G821 G822	
CHRONIC KIDNEY DISEASE	I120 I131 N03* N05* Z49* N18 N19 N25 N01 N074 N073 N072<br><br>N52 N19 N250 Z940 Z992	389500 394300 399501 549002 392701 392702 394200 549001 549012 549800 549801 549802 S22220 S22223 549800
DIABETES MELLITUS WITH COMPLICATIONS	E10* E11* E12* E13* E14* H360 H280 G590 G632 M142	

ANY TUMOR INCLUDING LEUKEMIA/LYMPHOMA (WITHOUT MALIGNANT SKIN NEOPLASIA)	C000 C001 C002 C003 C004 C005 C006 C007 C008 C009 C01X C020 C021 C022 C023 C024 C029 C030 C031 C039 C040 C041 C049 C050 C051 C052 C059 C060 C061 C062 C069 C07X C080 C081 C089 C090 C091 C099 C101 C102 C103 C104 C883 C887 C889 C900 C901 C91* C92* C93* C94* C95* C96*	
SEVERE LIVER DISEASE	K729 K766 K703 K767 K721	
METASTATIC SOLID TUMOUR	C780 C7* C8* C781 C782 C783 C784 C785 C786 C787 C788 C79 C790 C791 C792 C793 C794 C795 C796 C797 C798	
HIV	Z114 Z21X B200 B201 B202 B203 B204 B205 B206 B207 B208 B209 B210 B211 B212 B217 B219 B220 F028 R75X B220 B221 B24*	*ZIDOVUDINE* *LAMIVUDINE* *TENOFOVIR* *EMTRICITABIN* *DIDANOSINE* *NEVIRAPINE* *EFAVIRENZ* *ETRAVIRINE* *SAQUINAVIR* *LOPINAVIR* *ATAZANAVIR* *INDINAVIR* *NELFINAVIR* *RALTEGRAVIR* *ABACAVIR* *ABAMUNE* *RITONAVIR* *ATAZANAVIR*
NSAIDs		*DIFLUCINAL* *SULPARIDINA* *ANTIPIRINA* *FENASONA* *AMINOPIRINA* *DIPIRONA* *FENILBUTAZONA* *OXIFENBUTAZONA* *PIRAZINOBUTAZONA* *FEPRAZONA* *CLOFENAZONA* *BUMADIZONA* *SUXIBUZONA* *AZAPROPAZONA* *BENZIDAMINA* *SULINDAC* *DICLOFENAC* *ACECLOFENAC* *METIAZINICO* *ISONIXINA* *OXAPROXIN* *NIMESULIDA* *NEFOPAM* *ETODOLACO* *PIRROLACÉTICO* *KETOROLACO* *FENILACÉTICO* *DICLOFENACO* *IBUPROFEN* *KETOPROFEN* *NAPROXEN* *PIROXICAM* *TENOXICAM* *INDOLACÉTICO* *MEFENÁMICO* *PIRAZOLÓNICOS* *METAMIZOL* *CELECOXIB* *ROBECOXIB* *PARECOXIB* *ALDECOXIB* *ETORICOXIB* *NABUMETONA* *LUMIRACOXIB* *MELOXICAM* *CELECOXIB*
PNEUMONIA	J851 A403 J120 J128 J13X F060 J14X J150 J151 J152 J153 J154 J155 J156 J157 J158 J156 J159 J160 J168 J170 J171 J172 J180 J188 J189 B960	

Table S2. Adjusted odds ratios of AKI with RRT for patients using NSAIDs

Independen variable	OR (95%CI)	P value
NSAID	1.25 (1.12 - 1.40)	0.001
Male sex	1.54 (1.41 - 1.68)	0.001
Age group -----no. (%)		
40-49 yr.	1.23 (0.96 - 1.58)	0.101
50-59 yr.	1.20 (0.97 - 1.49)	0.092
60-69 yr.	1.44 (1.17 - 1.76)	0.001
70-79 yr.	1.76 (1.44 - 2.16)	0.001
>= 80 yr.	1.74 (1.41 - 2.14)	0.001
ICU admission		
Trauma	0.77 (0.64 - 0.92)	0.005
Sepsis	2.99 (2.70- 3.32)	0.001
Myocardial infarction	0.79 (0.70- 0.89)	0.001
Congestive heart failure	1.90 (1.58 - 2.28)	0.001
Contrast medium	1.44 (0.92 - 2.25)	0.106
Coexisting conditions __ no (%)		
Hypertension	1.94 (1.69 - 2.24)	0.001
Diabetes	1.99 (1.82 - 2.17)	0.001
Cerebrovascular disease	0.91 (0.77 - 1.07)	0.260
Chronic pulmonary disease	0.55 (0.50 - 0.61)	0.001
Connective tissue disease	0.79 (0.66 - 0.95)	0.011
Peptic ulcer disease	0.71 (0.61- 0.82)	0.001
Liver disease	2.28 (1.71 - 3.03)	0.001
Cancer	1.29 (0.99 - 1.66)	0.050

Table S3. Adjusted odds ratios of pneumonia for patients using NSAIDs

Independent variable	OR C.I (95%)	P value
NSAID	1.10 (1.00 - 1.20)	0.043
Male sex	1.17 (1.08 - 1.26)	0.001
Age group -----no. (%)		
40-49 yr.	1.04 (0.87 - 1.25)	0.635
50-59 yr.	0.91 (0.78 - 1.07)	0.262
60-69 yr.	0.80 (0.68 - 0.93)	0.005
70-79 yr.	1.05 (0.90 -1.21)	0.560
>= 80 yr.	1.66 (1.42-1.92)	0.001
ICU admission		
Trauma	0.82 (0.72 - 0.94)	0.005
Sepsis	37.00 (34.26 - 39.95)	0.001
Myocardial infarction	0.76 (0.68 - 0.85)	0.001
Congestive heart failure	1.24 (1.02 - 1.50)	0.001
Contrast medium	0.69 (0.46 - 1.05)	0.088
Coexisting conditions __ no (%)		
Hypertension	1.00 (0.90 - 1.10)	0.980
Diabetes	0.96 (0.88 - 1.04)	0.313
Cerebrovascular disease	1.37 (1.20 -1.56)	0.001
Chronic pulmonary disease	1.81 (1.68 - 1.97)	0.001
Connective tissue disease	1.11 (0.98 - 1.27)	0.098
Peptic ulcer disease	0.77 (0.68 - 0.86)	0.001
Liver disease	0.72 (0.49 - 1.06)	0.101
Cancer	1.41 (1.15 - 1.74)	0.001

Table S4. Adjusted odds ratios of mortality at 28 days for patients with acute kidney injury and RRT

Independent variable	OR C.I (95%)	P value
AKI with RRT	3.65 (3.21 - 4.02)	0.001
Male sex	1.15 (1.10 - 1.19)	0.001
Age group -----no. (%)		
40-49 yr.	1.26 (1.09 - 1.46)	0.001
50-59 yr.	1.54 (1.37 - 1.73)	0.001
60-69 yr.	2.28 (2.04 - 2.55)	0.001
70-79 yr.	3.73 (3.34 - 4.16)	0.001
>= 80 yr.	7.57 (6.79 - 8.45)	0.001
ICU admission		
Trauma	1.07 (0.99 - 1.16)	0.075
Sepsis	2.22(2.08 - 2.37)	0.001
Myocardial infarction	1.05 (1.03 - 1.11)	0.002
Congestive heart failure	1.94 (1.76 - 2.14)	0.001
Contrast medium	2.78 (2.7 - 3.40)	0.001
Coexisting conditions __ no (%)		
Hypertension	1.17 (1.09 - 1.25)	0.001
Diabetes	1.17 (1.12 - 1.22)	0.001
Cerebrovascular disease	1.47 (1.37 -1.58)	0.001
Chronic pulmonary disease	1.19 (1.14 - 1.25)	0.001
Connective tissue disease	1.01 (0.94 - 1.09)	0.763
Peptic ulcer disease	0.91 (0.85 - 0.97)	0.006
Liver disease	2.73 (2.35 - 3.20)	0.001
Cancer	2.64 (2.37 -2.93)	0.001
pneumonia	1.60 (1.47 -1.75)	0.001

Table S5: Baseline covariates

**Eligibility identifiers**			
**Covariate**	**Variable Name**	**Variable Type**	**Coding**
Participant ID	id	Numerical	Unique numerical identifier
Start of time interval	Date ICU admission	Continuous	Date ICU admission
End of time interval	28 days after ICU admission	Continuous	End of time interval
Age	age	Continuous	Years
	age_cat	Categorical	1: 18 – 39 years<br>2: 40 – 49 years<br>3: 50 – 59 years<br>4: 60 – 69 years<br>5: 70 – 79 years<br>6: >= 80 years
Sex assigned at birth	sex	Binary	0 : Female 1: Male
**Coexisting diseases**			
Hypertension	Hypertension before ICU admission	Binary	0: No 1: Yes
Diabetes	Diabetes before ICU admission	Binary	0: No 1: Yes
Cerebrovascular disease	Cerebrovascular disease before ICU admission	Binary	0: No 1: Yes
Chronic pulmonary disease	Chronic pulmonary disease before ICU admission	Binary	0: No 1: Yes
Connective tissue disease	Connective tissue disease before ICU admission	Binary	0: No 1: Yes
Peptic ulcer disease	Peptic ulcer disease before ICU admission	Binary	0: No 1: Yes
Liver disease	Liver disease before ICU admission	Binary	0: No 1: Yes
Cancer	Cancer before ICU admission	Binary	0: No 1: Yes
**ICU admission**			
Trauma	Trauma on ICU admission	Binary	0: No 1: Yes
Sepsis	Sepsis on ICU admission	Binary	0: No 1: Yes
Myocardial infarction	Myocardial infarction on ICU admission	Binary	0: No 1: Yes
Contrast medium	Contrast medium ` on ICU	Binary	0: No 1: Yes
**Treatment variable**			
nonsteroidal anti-inflammatory	NSAIDs	Binary	0: No use 1: Yes use
**Outcomes variables**			
Acute kidney injury with Replace Renal Treatment	AKI with RRT	Binary	0: No : 1: Yes
Pneumonia	Pneumonia	Binary	0: No : 1: Yes
Mortality	28 day mortality	Binary	0: No : 1: Yes

Figure S1. Graphical Abstract

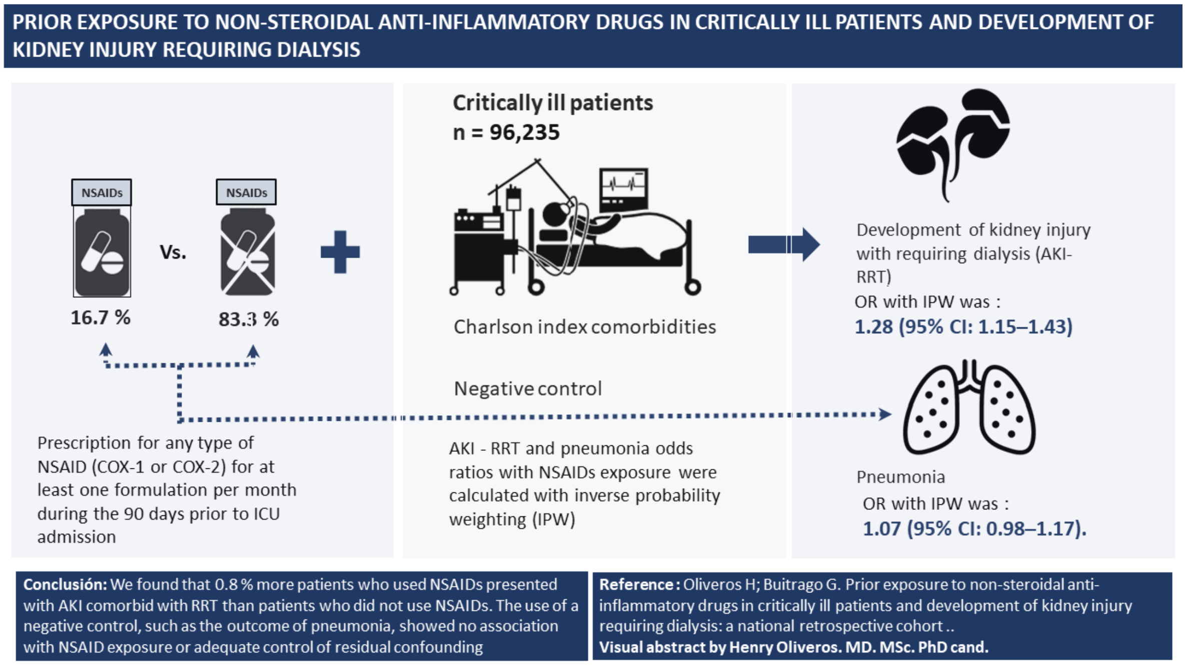

Figure S2. Distributions of the propensity index for users and non-users of NSAIDs.

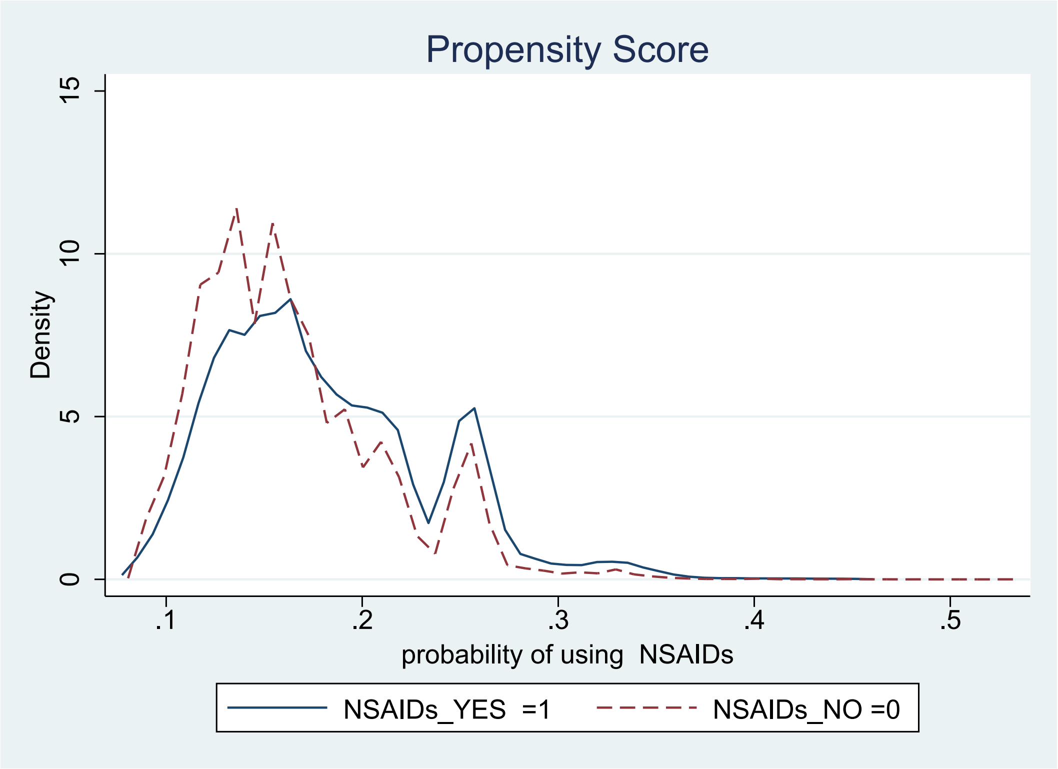

Figure S3. Factors for the development of mortality at 28 days

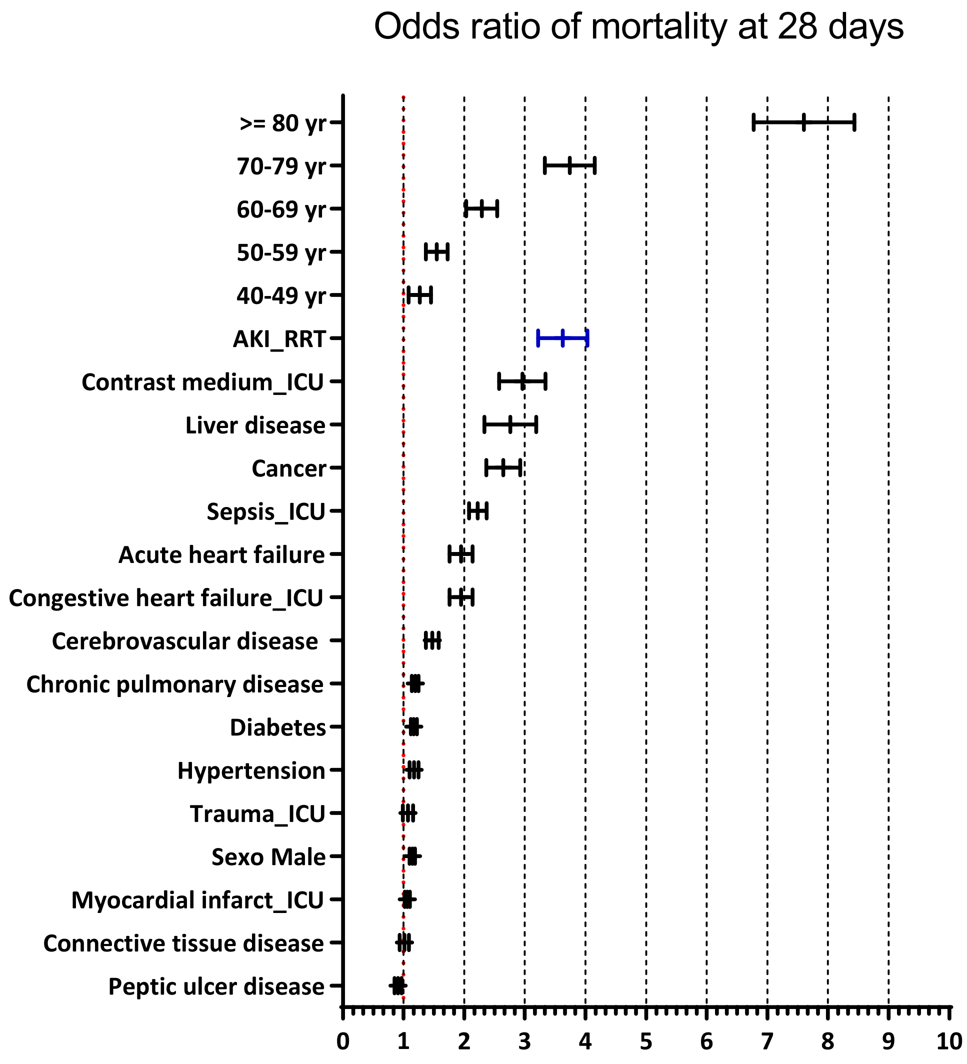

Figure S4. Comparative odds ratios between AKI and pneumonia associated with exposure to NSAIDs

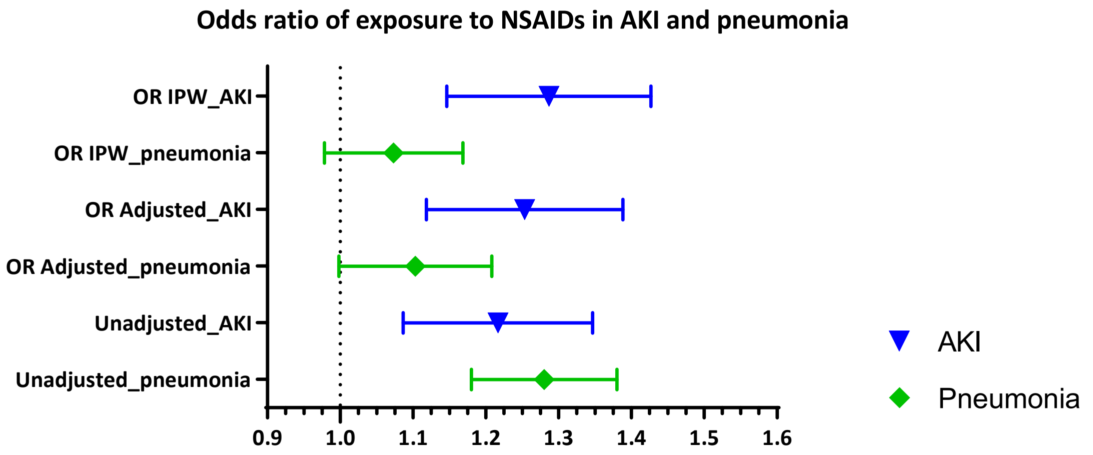

Adjusted by : Age, sex, trauma, sepsis, myocardial infarct, congestive heart failure, contrast medium, hypertension, diabetes, cerebrovascular disease, chronic pulmonary disease, peptic ulcer disease, liver disease, cancer.
